# Advances in the Preparation, Structure, and Biological Activity of Polysaccharides from *Nitraria* L.: A Review

**DOI:** 10.3390/foods15193518

**Published:** 2026-10-01

**Authors:** Bing Liu, Wenbei Dong, Guanrong Jia, Sayihan Bao, Dehui Yan

**Affiliations:** 1 School of Life Science and Engineering, Lanzhou University of Technology, Lanzhou 730050, China; 18219915618@163.com (W.D.); 19119390676@163.com (G.J.); ggbsyh1016@163.com (S.B.); 18794206016@163.com (D.Y.); 2 Gansu Industrial Technology Center for Food and Pharmaceutical Resources Development and Biomanufacturing, Lanzhou 730050, China

**Keywords:** *Nitraria* L. polysaccharides, extraction and separation, chemical structure, biological activity, mechanism of action

## Abstract

*Nitraria* L. species are drought- and salt-tolerant plants whose fruits, and in some studies leaves, have been investigated as sources of bioactive polysaccharides. However, the reported polysaccharide preparations and purified fractions are chemically heterogeneous and originate from different *Nitraria* species, geographic regions, plant organs, and extraction and fractionation procedures. This review critically summarizes current knowledge of the raw materials, extraction and purification methods, structural characteristics, and biological activities of *Nitraria*-derived polysaccharides. Particular attention is given to the relationship between extraction/fractionation procedures and the polysaccharide populations recovered, because extraction conditions may influence not only yield but also molecular weight, monosaccharide composition, branching, and other structural features. Reported fractions show substantial structural diversity in molecular-weight distribution, monosaccharide composition, and, where sufficiently characterized, glycosidic-linkage patterns and polysaccharide class, while differences in analytical procedures further limit direct cross-study comparisons. Biological studies have reported antioxidant, immunomodulatory, anti-inflammatory, antitumor, hypoglycaemic, anti-fatigue, anti-stress, hepatoprotective, hypolipidemic, analgesic, organ-protective, and gut-microbiota-modulating effects. Nevertheless, these findings were obtained using structurally distinct preparations and heterogeneous experimental systems, doses, administration routes, and endpoints; therefore, they should not be regarded as uniform properties of *Nitraria* polysaccharides as a whole. Current within-study comparisons suggest that fractionation and chemical modification may influence biological activity, but the available evidence remains insufficient to establish general structure–activity relationships. Future studies should prioritize standardized raw-material characterization, extraction and fractionation, detailed structural analysis, and biological evaluation of well-defined polysaccharide fractions to establish more reliable structure–activity relationships and support their rational development as functional-food ingredients and related bioactive products.

## 1. Introduction

*Nitraria* L. is a xerophilous or hyperxerophilous deciduous shrub belonging to the genus *Nitraria* in the family Zygophyllaceae, commonly known as “desert cherry”. It is distributed across Asia, Europe, Africa, and Australia, and is typically found in saline–alkali environments, arid deserts, sandy lake basins, slopes, and lowlands [1,2]. China is the primary distribution area for plants of the genus *Nitraria*. Currently, six species of *Nitraria* have been identified within China, including *Nitraria sibirica* Pall, *Nitraria sphaerocarpa* Maxim., *Nitraria roborowskii* Kom., *Nitraria tangutorum* Bobrov, *Nitraria pamirica* L.I.Vassiljeva, and *Nitraria schoberi* L. Tolerant of drought and high salinity, it can adjust its leaf ultrastructure and regulate metabolism under alkaline stress to adapt to extreme saline–alkali environments, making it an excellent sand-fixing vegetation for saline–alkali lands [3]. Such adaptation to arid and saline environments may influence carbon allocation and cell-wall polysaccharide biosynthesis, potentially contributing to provenance-dependent variation in polysaccharide composition and structure. However, direct evidence linking habitat conditions to the biosynthesis of *Nitraria*-derived polysaccharides remains limited. *Nitraria* is primarily distributed in the Xinjiang Uygur Autonomous Region, Gansu Province, Inner Mongolia Autonomous Region, Ningxia Hui Autonomous Region, Shaanxi Province, Qinghai Province, and Tibet Autonomous Region. Species of the genus *Nitraria* are drought-tolerant and resistant to wind and sand, making them excellent sand-fixing plants as well as a source of fodder in arid and semi-arid regions [4]. As a plant that combines regional distinctiveness with practical value, *Nitraria* has a long history of use in folk traditions. *Nitraria* is also a traditional source of ethnobotanical medicine; records of its fruits being used medicinally are found in Medicinal Plants of China’s Desert Regions and they are primarily used in folk medicine. According to Compendium of Materia Medica, *Nitraria* is pungent in flavour, cold in nature, and non-toxic. It can relieve abscesses, facilitate pus discharge, tonify kidney qi, and replenish jing and marrow according to traditional Chinese medicine, and is traditionally used for the treatment of spleen–stomach weakness, dyspepsia, neurasthenia, and dizziness. In The Great Dictionary of China, *Nitraria* is referred to as “Kami.” The fruits are harvested when ripe, sun-dried for medicinal use, and possess the functions of strengthening the spleen and stomach, tonifying and fortifying the body, and regulating menstruation while promoting blood circulation. It is primarily used to treat physical weakness, functional disorders of the spleen and stomach, indigestion, irregular menstruation, and lower back pain associated with a deficiency cold pattern [5].

**Figure 1:**
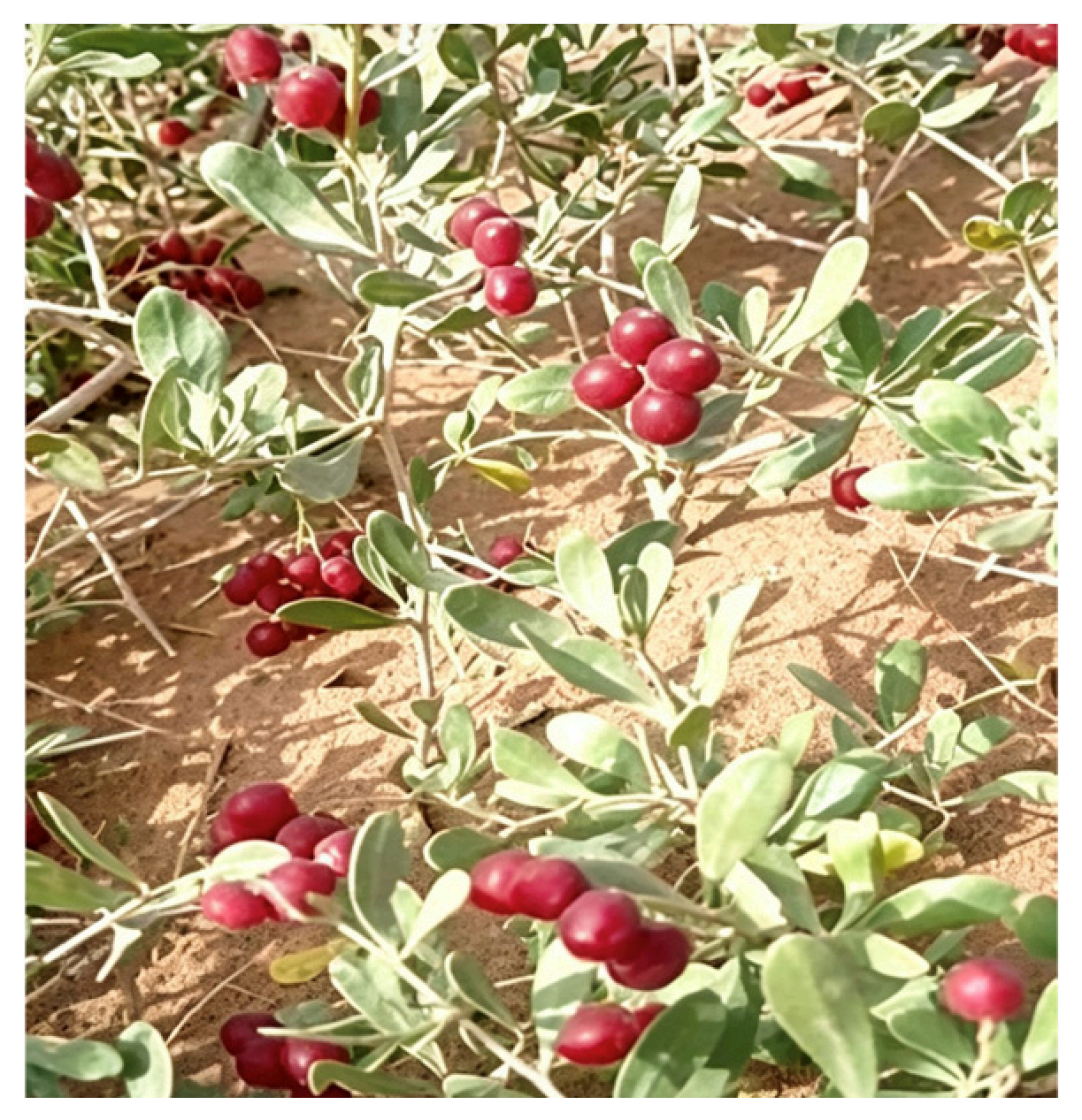
Images of *Nitraria* plants and fruits. Taken in Minqin County, Wuwei City, Gansu Province in 2025.

Current studies have reported that the active ingredients in *Nitraria* fruits include polysaccharides, flavonoids, alkaloids, amino acids, and trace elements [6]. Flavonoids derived from *Nitraria* can increase the carbon clearance index in mice and effectively improve and enhance the humoral immune function of mice with cyclopiazonic acid-induced immune deficiency [7]. The fruits of *Nitraria* contain at least five categories of anthocyanin derivatives, namely pelargonidin, delphinidin, cyanidin, malvidin, and peonidin. These anthocyanins possess lipid-lowering, anti-atherosclerotic, and antioxidant activities [8]. Anthocyanin extracts from *Nitraria* exert neuroprotective effects by reducing the levels of reactive oxygen species and malondialdehyde (MDA) in the hippocampal tissue of rats, thereby alleviating D-galactose-induced memory impairment in rats [9]. The alkaloids isolated from *Nitraria* plants are mainly classified into two basic structural types: β-carboline and quinazoline. The major alkaloids identified from *Nitraria* include tangutorine, nitraine, nitarmine, isonitramine, and schoberine. Among them, tangutorine is a novel alkaloid with a unique chemical structure. *In vitro* studies have verified that low-dose tangutorine can slightly stimulate the proliferation of human colon cancer HT-29 cells [10]. *Nitraria* fruits are rich in protein and contain a complete spectrum of amino acids, with essential amino acids accounting for 40% of the total amino acid content. The contents of glutamic acid, arginine, aspartic acid, and alanine in *Nitraria* fruits all exceed 1%, and glutamic acid is the most abundant amino acid component [11]. Thirteen trace elements have been detected in *Nitraria* fruits, among which iron (Fe), calcium (Ca), potassium (K), and sodium (Na) are the four most predominant elements, with a total content of 22.676 mg/100 g [12]. Selenium (Se) has also been detected in the fruits of *Nitraria*. As a key component of glutathione peroxidase, selenium can enhance the antioxidant capacity of the organism and prevent oxidative stress-related diseases [13]. An overview of the main characteristics of *Nitraria* is shown in Figure 1.

Research on *Nitraria* fruit polysaccharides has mainly addressed extraction and purification, structural characterization, and *in vitro* and *in vivo* biological activities. Modern pharmacological studies have demonstrated that polysaccharides are important bioactive constituents of *Nitraria*. In this review, the structurally diverse polysaccharide preparations and purified fractions obtained from *Nitraria* species are collectively discussed as *NPS* while recognizing that they represent chemically distinct polysaccharides rather than a single structural class. This review systematically summarizes current research on NPSs, focusing on their extraction and preparation, structural characteristics, biological activities, and proposed mechanisms of action. The raw-material sources and polysaccharide preparations investigated in the available studies are summarized in Table 1.

## 2. Extraction and Isolation

**Figure 2:**
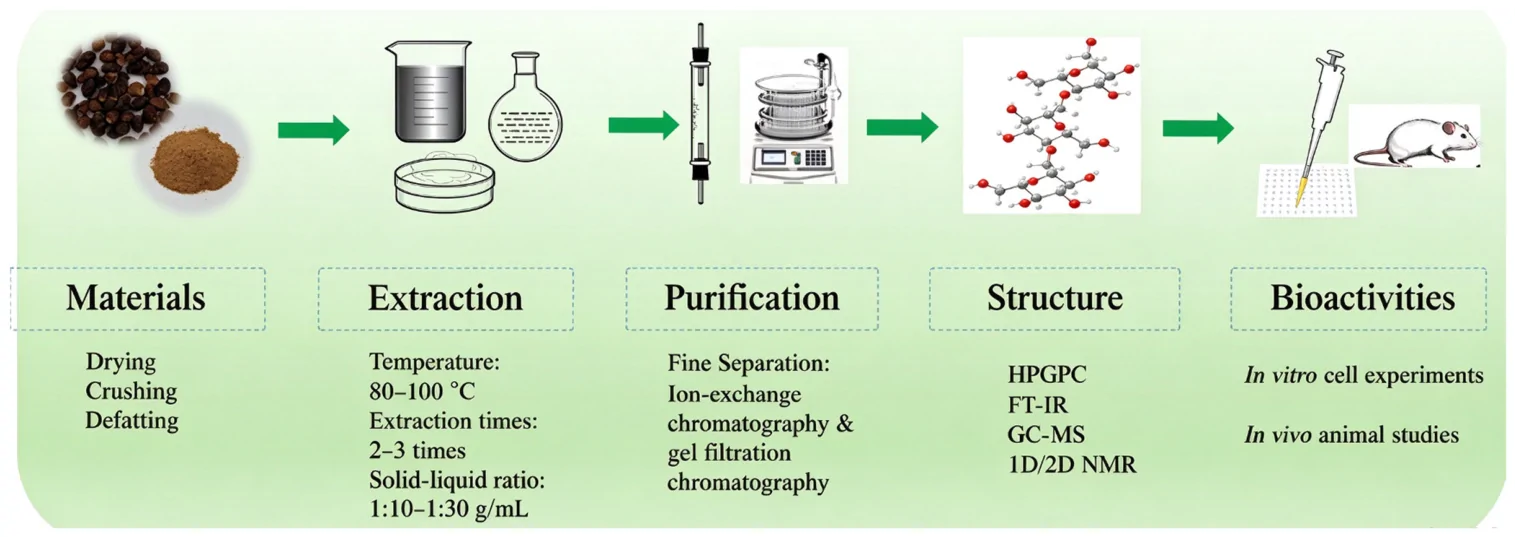
Flow chart of *Nitraria* L. polysaccharides extraction.

Polysaccharides are the primary bioactive constituents of *Nitraria* fruit. Their extraction, separation, and purification are essential prerequisites for subsequent structural elucidation and biological activity evaluation. Hot-water extraction is the most widely used method to prepare water-soluble *Nitraria* polysaccharides. Raw materials are pretreated by drying, crushing, and defatting to remove impurities and enhance extraction efficiency. The obtained crude polysaccharides are sequentially purified: deproteinization and depigmentation are performed to remove proteins and pigments, followed by fine separation using ion-exchange and gel filtration chromatography to obtain homogeneous polysaccharide fractions. Structural characterization and bioactivity studies can be further carried out on purified polysaccharides. The overall technical route is shown in Figure 2.

### 2.1. Extraction of NPSs

Published studies have generally prepared *Nitraria* fruit polysaccharides by washing, low-temperature drying, grinding, and sieving the fruits, followed by defatting with petroleum ether and, where applicable, ethanol decolorization. The pretreated white thorn fruit powder was extracted in water; the supernatants were combined, concentrated under vacuum to 1/5 of the original volume, precipitated with 4 times the volume of 95% ethanol, and freeze-dried [14,17]. Various extraction approaches, including conventional hot-water extraction, water extraction–ethanol precipitation, enzyme-assisted extraction, and ultrasound–microwave-assisted extraction, have been applied to recover *NPSs*. These methods may differ not only in extraction yield but also in the composition and structural characteristics of the resulting polysaccharide fractions (Table 2).

Extraction conditions may influence not only polysaccharide yield but also the composition and structural characteristics of the polysaccharide fractions recovered from plant materials. Different extraction systems can preferentially release different cell-wall polysaccharide populations, and variations in extraction solvent, temperature, and treatment intensity may consequently affect molecular weight, monosaccharide composition, branching, and other structural features [29,30]. For example, the ammonium oxalate-assisted extraction reported for *Nitraria* leaves facilitates the release of cell-wall-associated pectic polysaccharides [16], consistent with the ability of oxalate to chelate divalent cations and solubilize pectins associated with calcium cross-links [31]. Hot-water extraction mainly recovers water-soluble polysaccharide fractions, whereas acid-, alkali-, and enzyme-assisted procedures may release different cell-wall components and generate polysaccharide fractions with different structural profiles [29,30]. Therefore, the structural differences observed among reported *Nitraria*-derived polysaccharide fractions may reflect not only differences in species, plant organs, and geographic origin, but also the influence of extraction and subsequent fractionation procedures. These methodological factors should therefore be considered when comparing structural characteristics across studies.

In addition to the target polysaccharides, the NPS also contains proteins, pigments, monosaccharides, and other small-molecule substances. Therefore, the polysaccharide extract requires further purification through methods such as ethanol precipitation, deproteinization, and decolorization. Ethanol precipitation of the NPS involves first concentrating the polysaccharide extract to a suitable volume and then adding ethanol. Since polysaccharides have poor solubility in ethanol, they precipitate out, separating them from other water-soluble impurities. The Sevag method is commonly used for deproteinization. A 20% solution of Sevag reagent (chloroform:n-butanol = 5:1, v/v) was added to a crude polysaccharide solution from Nitraria; the resulting mixture was vigorously shaken at 25 °C for 30 min and then centrifuged for 15 min. The upper polysaccharide solution was then collected. This procedure was repeated 3–5 times until no significant precipitate remained in the middle layer. The deproteinization rate reached a 65–70% range, with a polysaccharide loss rate range of only 5–8% [14]. A 5% (m/m) trichloroacetic acid (TCA) solution was added dropwise to the crude polysaccharide solution; the pH was adjusted to a range of 2.5–3.0; the solution was incubated in a constant-temperature water bath for a 30–40 min range, then centrifuged to collect the supernatant; the pH was adjusted back to neutral with 1 mol/L NaOH, activated carbon was then added, followed by stirring and filtration; the deproteinization rate can reach a 70.96–75% range [17]. Pigments co-extracted during *Nitraria* polysaccharide preparation include water-soluble anthocyanins, such as pelargonidin-, delphinidin-, cyanidin-, malvidin-, and peonidin-derived pigments reported in *Nitraria* fruits [9]. Common decolorization methods include activated carbon adsorption, hydrogen peroxide oxidation, and macroporous adsorption resin adsorption. The activated carbon adsorption method achieves high decolorization efficiency but results in a relatively high polysaccharide loss rate; the hydrogen peroxide oxidation method tends to oxidize the hydroxyl groups of polysaccharides, thereby destroying their structural activity; and the macroporous adsorption resin adsorption method balances decolorization efficiency with polysaccharide retention and is therefore the preferred approach. A comparison of 10 types of macroporous adsorption resins, including AB-8, HPD-100, and HPD-100A, revealed that the HPD-100A resin exhibited the best overall performance, with a decolorization rate of 74.78% and a polysaccharide recovery rate of 86.31% [17].

### 2.2. Purification of NPSs

Currently, the mainstream media used in laboratories for the isolation of white thorn polysaccharides are the DEAE series of anion-exchange media (DEAE-Cellulose, DEAE Sepharose Fast Flow) and Sephadex gel filtration resin [18,21]. DEAE chromatography separates neutral and acidic polysaccharides based on charge differences, while gel filtration chromatography separates components according to molecular weight. The crude hot-water-extracted polysaccharides from Nitraria tangutorum fruits (NTWPs) were eluted using a 0–1.0 mol/L NaCl gradient range on a DEAE-52 column; the major polysaccharides were then redissolved in deionized water and further purified on a G-200 column to yield the purified polysaccharide NTWP-II [27]. The *Nitraria* tangutorum-derived polysaccharide preparation (NTP) was purified by DEAE-52 ion-exchange chromatography; elution with distilled water and 0.3 mol/L NaCl yielded two fractions, NTP-I and NTP-II [21]. Polysaccharides from *Nitraria retusa* Pall. were purified using DEAE- Sepharose FF and eluted with deionized water, 0.1 mol/L NaCl, and 0.3 mol/L NaCl to yield three fractions: NRFP-1, NRFP-2, and NRFP-3. The recovery rates for these three fractions were 16.23%, 15.84%, and 12.56% [22]. The crude polysaccharide NSP from *Nitraria sibirica* Pall. was similarly purified using DEAE cellulose 52; fractions NSP-1, NSP-2, and NSP-3 were collected by elution with distilled water, and 0.2 and 0.4 mol/L NaCl gradients [15]. After freeze-drying the precipitate to obtain NTWP, two polysaccharide fractions (NTWP-N and NTWP-A) were eluted from a DEAE cellulose column. The freeze-dried NTWP-A was eluted using a DEAE agarose rapid-flow column with a linear gradient range of 0–0.5 mol/L NaCl, ultimately yielding NTWP-Ap [18].

## 3. Structural Characteristics

The structural characteristics reported for *Nitraria* polysaccharides include molecular weight, monosaccharide composition, functional groups, glycosidic-linkage patterns, and, in some studies, higher-order conformation. Because the reported NPS fractions were obtained from different *Nitraria* species, geographic origins, plant organs, and extraction/fractionation procedures, their structural parameters vary substantially among studies and should be interpreted at the level of individual fractions rather than as common characteristics of a single polysaccharide entity. The following subsections therefore summarize the molecular-weight characteristics, monosaccharide compositions, and primary structural features of individual *Nitraria*-derived polysaccharide fractions. The available structural information is compiled in Table 3.

### 3.1. The Molecular Weight of NPSs

Reported *Nitraria*-derived polysaccharide preparations and fractions exhibit a broad range of apparent molecular weights, ranging from approximately 1000 Da to more than 1,000,000 Da. Considerable variation has been reported among individual fractions. For example, NTWP contains components with molecular weights greater than 1,000,000 Da [19,20], whereas NRFP-3 and CMNTP have reported molecular weights of 67,450 Da [22] and up to 80,385 Da [17], respectively, and NSP-C has a much lower molecular weight of 5070 Da [15]. Such variation may reflect not only intrinsic differences among polysaccharide fractions, but also differences in extraction severity, processing-related degradation, chemical or enzymatic modification, and subsequent fractionation [29,30]. In addition, the apparent molecular-weight values may be influenced by differences in chromatographic systems, calibration standards, and other analytical conditions. Therefore, molecular-weight values reported in different studies should be interpreted in the context of the corresponding preparation and analytical procedures rather than regarded as directly comparable characteristics of a single polysaccharide class. A major limitation of the current literature is the lack of standardized extraction, fractionation, and molecular-weight determination procedures, which restricts direct quantitative comparison among reported fractions.

### 3.2. The Monosaccharide Composition of NPSs

NPSs are all heteropolysaccharides composed of 5 to 10 types of monosaccharides, with glucose (Glc), galactose (Gal), arabinose (Ara), and rhamnose (Rha), and are commonly accompanied by mannose (Man), xylose (Xyl), and fucose (Fuc). The acidic components additionally contain glucuronic acid (GlcUA) and galacturonic acid (GalUA). There are significant differences in the proportions of these monosaccharides across different regions and varieties [14,17,21]. In *Nitraria* polysaccharides produced in provinces of Northwest China, glucose (Glc), galactose (Gal), and arabinose (Ara) constitute the majority [32], *Nitraria* polysaccharides in Hebei Province, China, have a relatively high proportion of mannose (Man) [25], while those from the Gafsa region are characterized by high levels of uronic acids, with glucose (Glc) accounting for 41.4% and galacturonic acid (GalUA) for 30.5%; these are the key features that distinguish them from *Nitraria* polysaccharides in China [23]. However, these compositional differences should not be attributed solely to geographic origin, because the reported studies also differ in species, plant organ, extraction/fractionation procedures, and analytical methods.

### 3.3. The Primary Structure of NPSs

The type, position, and configuration of the glycosidic bonds directly determine the chain structure and spatial conformation of white thorn polysaccharides; their core characteristics were identified using techniques such as nuclear magnetic resonance (NMR), periodate oxidation, Smith degradation, and FT-IR. The linkage patterns and terminal structures of glycosidic bonds in carbohydrate chains are also important factors affecting molecular recognition. Related studies, through the construction of specific glycan structures and analysis of their interactions with lectins, have demonstrated that fine glycan structures are closely associated with molecular recognition behaviour [33]. *Nitraria* polysaccharides primarily exhibit a mixed α- and β-configuration, with a small number having a single configuration: α-glycosidic bonds exhibit characteristic peaks at a range of 830–850 cm^−1^ in FT-IR, with terminal proton chemical shifts in the range of 4.8–5.2 ppm in NMR; for example, NRLP produced by Gafsa contains only α-glycosidic bonds [24]; and β-type glycosidic bonds exhibit characteristic peaks at a 880–890 cm^−1^ range with a chemical shift range of 4.4–4.8 ppm; for example, NRFP from Gafsa consists solely of β-type bonds [23]. The vast majority of polysaccharides contain both configurations simultaneously, such as NTP-I from Wuwei, Gansu [21], and NRFP from Baicheng, Xinjiang [22], where the main chain is predominantly β-type and the side chains are predominantly α-type. NPS exhibits a rich variety of linkage patterns; the main chain consists primarily of β-type linkages, with the core including β-(1→3), β-(1→4), α-(1→4), and α-(1→6); side chains are predominantly α-type, including α-(1→5) (characteristic Ara linkage), α-(1→2) (Rha linkage), and α-(1→6) (Glc and Gal linkages), with branching points primarily occurring at the O-2, O-3, and O-6 positions [15,27]. The linkage sequences of some high-purity polysaccharides have been elucidated. For example, the main chain of NTWP-II consists of (1→3)-β-Gal, with side chains linked via (1→3) to α-Man and α-Ara, and via (1→6) to α-Glc [27]; NSP-C contains various linkages, including 1→2, 1→4, and 1→6, with Gal linked to the main chain via glycosidic bonds that are not oxidized by periodate [14].

## 4. Bioactivities

Biological activities have been evaluated for chemically distinct *Nitraria*-derived crude polysaccharide preparations and purified fractions using heterogeneous *in vitro* and *in vivo* experimental systems. Reported effects include antioxidant, immunomodulatory, anti-inflammatory, antitumor, hypoglycaemic, anti-fatigue, anti-stress, hepatoprotective, analgesic, organ-protective, and gut-microbiota-modulating activities. Table 4 summarizes these findings at the level of the tested preparation or fraction, together with its source, structural class where available, experimental system, and dose or concentration. Because the reported studies differ in species, plant organ, fraction composition, structural characteristics, experimental model, administration route, dose, and biological endpoint, the observed effects should not be interpreted as uniform properties of *Nitraria*-derived polysaccharides as a whole. In particular, *in vitro* concentrations and *in vivo* doses are not directly comparable.

Within this heterogeneous evidence base, only limited conclusions can currently be drawn regarding structure–activity relationships. Within-study comparisons are generally more informative than comparisons across different reports. For example, carboxymethylation of NTP to CMNTP altered its molecular-weight distribution and chemical characteristics and was accompanied by enhanced hydroxyl- and superoxide-anion-scavenging activities [17], suggesting that chemical modification may influence antioxidant performance. NTP-I is a relatively low-molecular-weight fraction and exhibits both antioxidant and hypoglycaemic activities [21]; however, these observations alone do not establish low molecular weight as the determinant of activity. Similarly, NRFP-1, NRFP-2, and NRFP-3 [22], as well as NSP-1, NSP-2, and NSP-3 [15], differ in molecular weight and monosaccharide composition and also exhibit differences in biological activity. Because multiple structural variables change simultaneously among these fractions, the individual contributions of molecular weight, uronic-acid content, monosaccharide composition, branching, and polysaccharide class cannot yet be clearly separated. Therefore, the available evidence is insufficient to establish a general structure–activity relationship for *Nitraria*-derived polysaccharides.

### 4.1. Antioxidant Activity

**Figure 3:**
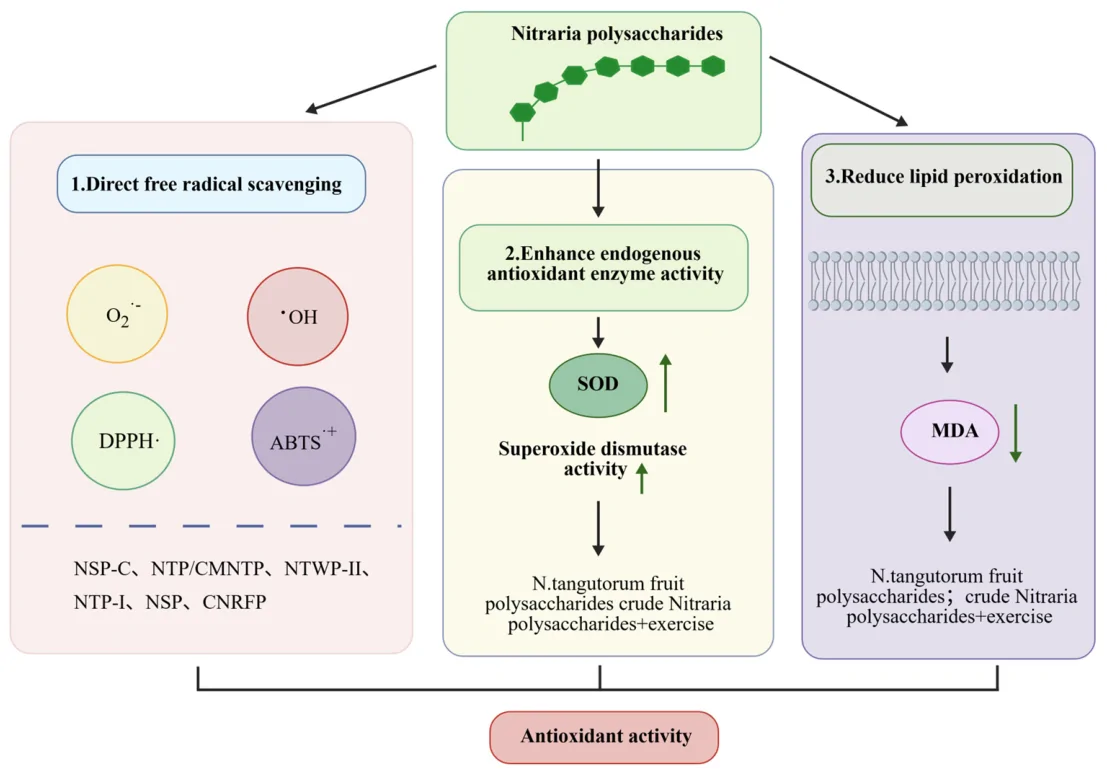
The potential antioxidant activity of *Nitraria* L. polysaccharides. Upward arrows (↑) indicate an increase or upregulation, downward arrows (↓) indicate a decrease or downregulation, and connecting arrows indicate the proposed relationships or effects.

Reactive oxygen species (ROS) are continuously generated during cellular metabolism. The endogenous antioxidant system, including antioxidant enzymes such as superoxide dismutase (SOD), catalase (CAT), and glutathione peroxidase (GPx), as well as non-enzymatic antioxidants such as glutathione and vitamins, plays an important role in maintaining redox homeostasis. Excessive accumulation of ROS can result from both endogenous disturbances, such as mitochondrial dysfunction and inflammatory responses, and exogenous stressors, leading to lipid peroxidation and damage to proteins, lipids, and nucleic acids, and long-term redox imbalance is prone to induce metabolic disorders, cardiovascular diseases, tumours, and multiple other illnesses [17,24]. Purified polysaccharide NSP-C from *Nitraria sibirica* features a low molecular weight and a triple-helix spatial conformation, exhibiting a prominent *in vitro* antioxidant capacity, with IC50 values of 0.209 mg/mL for 2,2-diphenyl-1-picrylhydrazyl (DPPH) radicals and 0.149 mg/mL for hydroxyl radicals [14]. Both the *Nitraria tangutorum* polysaccharide (NTP) and its carboxymethylated derivative CMNTP possess *in vitro* antioxidant activity. At 0.2 mg/mL, their hydroxyl-radical scavenging rates reached 17.97% and 29.61%, respectively. At 0.4 mg/mL, superoxide-anion scavenging rates were 3.97% and 9.53%. At 1.0 mg/mL, their DPPH-radical scavenging rates were 37.94% and 12.10%. These results indicate that carboxymethylation enhances the scavenging capacity against hydroxyl radicals and superoxide anions but reduces DPPH-radical scavenging activity. The purified polysaccharide NTWP-II displayed favourable scavenging effects on hydroxyl radicals and superoxide anions [17]. The low-molecular-weight *Nitraria tangutorum* polysaccharide NTP-I achieved a DPPH-radical scavenging rate of 71.72 ± 2.1% at 1 mg/mL [21]. Crude polysaccharide NSP from N. sibirica exerted dose-dependent scavenging effects on DPPH radicals, the 2,2′-azino-bis(3-ethylbenzothiazoline-6-sulfonic acid) radical cation (ABTS•+), and hydroxyl radicals, with corresponding IC_50_ values of 0.743 mg/mL, 0.183 mg/mL, and 0.604 mg/mL [15]. Fruit polysaccharides of *Nitraria tangutorum* exhibited satisfactory *in vivo* antioxidant effects in mice. After treatment with high- dose and low-dose polysaccharides (3 g/kg and 1.5 g/kg), serum SOD activity increased from 161.1 ± 12.13 U/mL in the normal control group to 179.4 ± 6.07 U/mL and 178.5 ± 12.57 U/mL, respectively. Meanwhile, MDA contents decreased from 6.44 ± 0.58 nmol/mL to 5.47 ± 0.64 nmol/mL and 5.73 ± 0.63 nmol/mL. This demonstrates that the polysaccharides can boost the activity of antioxidant enzymes and alleviate lipid-peroxidation-mediated damage *in vivo* [34]. Combined supplementation of crude *Nitraria* polysaccharides and progressive-load aerobic exercise significantly improved myocardial antioxidant capacity in ageing rats. After oral administration of *Nitraria* polysaccharides at doses of 100, 200, and 300 mg/(kg·d), myocardial SOD activities reached 526.29 ± 95.29 U/mg, 561.59 ± 101.54 U/mg, and 602.68 ± 93.28 U/mg, while MDA contents dropped to 1.18 ± 0.31 nmol/mg, 0.99 ± 0.16 nmol/mg, and 0.93 ± 0.24 nmol/mg, all significantly different from the exercise-only group (*p* < 0.01). The finding suggests that *Nitraria* polysaccharides combined with aerobic exercise can elevate antioxidant-enzyme activity and mitigate lipid-peroxidation injury [35]. *In vitro* radical scavenging assays have been performed on polysaccharides from *Nitraria retusa* [22], *Nitraria* tangutorum [28], *Nitraria roborowskii* [32], *Nitraria retusa* [23,24], as well as N. *sibirica* polysaccharides subjected to different drying procedures [36]. All these polysaccharides showed radical scavenging and reducing capacities to varying degrees. Among them, fruit polysaccharides of *Nitraria tangutorum* had IC_50_ values of 0.08854 mg/mL for DPPH radicals and 2.5492 mg/mL for hydroxyl radicals. Crude *Nitraria* roborowskii fruit polysaccharide (CNRFP) exhibited dose-dependent scavenging activity toward DPPH radicals, ABTS•+ radical cations, and hydroxyl radicals, with IC_50_ values of 1.14 mg/mL, 0.54 mg/mL, and 1.11 mg/mL, respectively. Different drying and decolorization treatments altered the antioxidant performance of N. sibirica polysaccharides. The enhanced radical scavenging activity observed in some decolourised samples may be related to changes in polysaccharide purity and composition caused by the treatment process; however, the underlying mechanism was not clarified in the original study. Collectively, *Nitraria* polysaccharides exert favourable antioxidant activities both *in vitro* (radical scavenging) and *in vivo*. Their antioxidant mechanisms mainly include scavenging reactive-oxygen-species radicals, enhancing endogenous antioxidant-enzyme activities and alleviating lipid-peroxidation damage. Hence, *Nitraria* polysaccharides hold great potential for development as natural antioxidant raw materials (Figure 3).

### 4.2. Immunomodulatory and Anti-Inflammatory Activities

**Figure 4:**
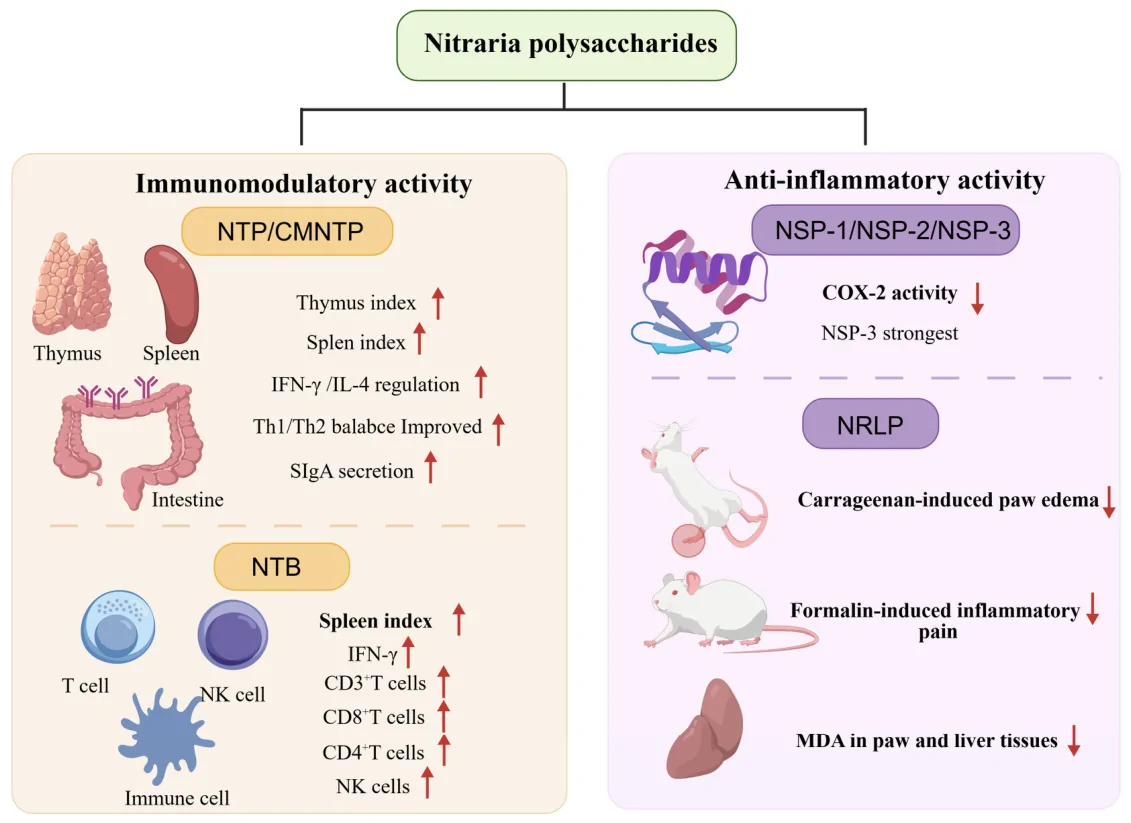
The potential immunomodulatory activity of *Nitraria* L. polysaccharides. Upward arrows (↑) indicate an increase or upregulation, downward arrows (↓) indicate a decrease or downregulation, and connecting arrows indicate the proposed relationships or effects.

Immunomodulation and inflammatory responses synergistically maintain organismal homeostasis. Imbalances in immune organs, immune cells, and cytokines not only weaken host immune defence capacity, but are also accompanied by inflammatory reactions and oxidative damage. Studies have shown that both polysaccharide NTP from *Nitraria tangutorum* and its carboxymethylated derivative CMNTP can ameliorate immune function in cyclophosphamide-induced immunosuppressed mice [17]. At the dosage of 200 mg/kg, the thymus indices of NTP and CMNTP groups were 0.393 ± 0.106% and 0.470 ± 0.094%, while the spleen indices reached 0.352 ± 0.056% and 0.501 ± 0.118%, respectively. Both NTP and CMNTP increased serum IFN-γ and IL-4 levels, alleviated Th1/Th2 immune imbalance, and promoted secretory immunoglobulin A (SIgA) secretion in the intestinal mucosa; these immunomodulatory effects were generally stronger after carboxymethylation. Further research showed that administration of 3.6 g/kg NTB polysaccharide increased the spleen index of immunosuppressed mice from 1.73 ± 0.17 to 3.18 ± 0.03; the relative expression level of IFN-γ/β-actin rose from 0.267 ± 0.225 to 0.545 ± 0.227 [37]. The proportions of peripheral blood CD3^+^, CD4^+^, CD8^+^, and NK cells increased from 38.6 ± 8.24%, 13.5 ± 9.07%, 11.1 ± 8.86%, and 10.8 ± 2.04% to 67.4 ± 13.06%, 31.6 ± 2.30%, 17.6 ± 6.42%, and 20.7 ± 1.03%, respectively. Meanwhile, serum IFN-γ concentration was significantly elevated. In addition, *Nitraria sibirica* polysaccharides NSP-1, NSP-2, and NSP-3 at 100 μg/mL exhibited COX-2 inhibitory rates of 79.6%, 54.7%, and 85.6%, among which NSP-3 possessed the strongest inhibitory activity [15]. Water-soluble leaf polysaccharide NRLP from *Nitraria retusa* exerted maximum inhibition rates of 68.0% and 71.9% against carrageenan-induced paw oedema at doses of 100 mg/kg and 400 mg/kg, respectively. At 400 mg/kg, it achieved a 76.4% inhibition rate in the late phase of formalin-evoked inflammatory pain, and reduced MDA contents in paw pad and liver tissues [24]. Collectively, *Nitraria* polysaccharides exert multi-layered regulatory effects on immune-inflammatory homeostasis. They can improve the status of immune organs; modulate T lymphocytes, NK cells, as well as immune cytokines including IFN-γ and IL-4; and enhance intestinal mucosal immunity. Meanwhile, they suppress COX-2 activity, relieve inflammatory oedema, and mitigate inflammation-associated oxidative damage. Therefore, *Nitraria* polysaccharides exhibit promising exploitation potential as natural raw materials with both immunomodulatory and anti-inflammatory activities (Figure 4).

### 4.3. Antitumor Activity

**Figure 5:**
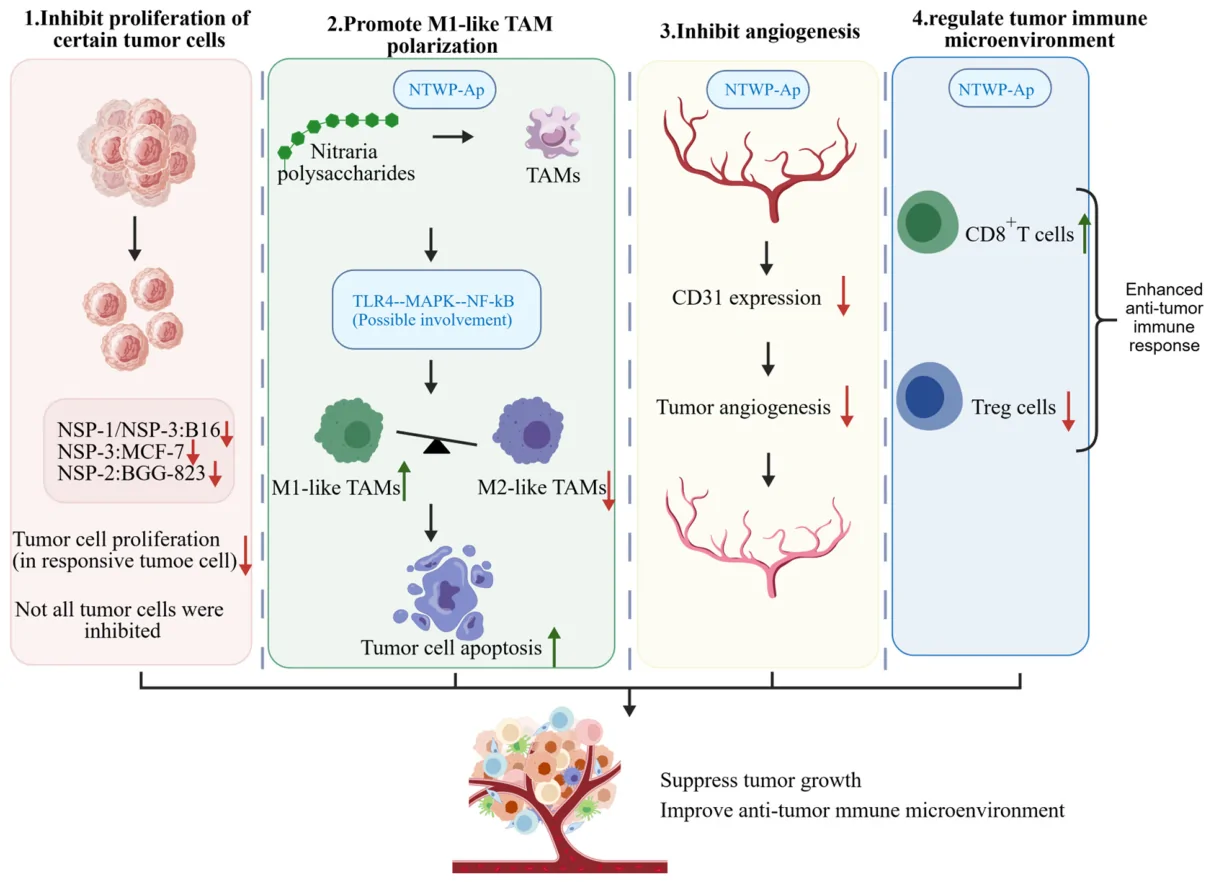
The potential antitumor activity of *Nitraria* L. polysaccharides. Upward arrows (↑) indicate an increase or upregulation, downward arrows (↓) indicate a decrease or downregulation, and connecting arrows indicate the proposed relationships or effects.

Carcinogenesis is a multistep process involving initiation, promotion, and progression. During these stages, genetically or epigenetically altered cells undergo clonal expansion and progressively acquire malignant characteristics. Subsequent tumour growth and progression are further influenced by pathological angiogenesis and interactions within the tumour-immune microenvironment. Antitumor bioactivities mainly include inhibiting tumour-cell proliferation, inducing cell apoptosis, suppressing angiogenesis, and remodelling the tumour-immune microenvironment. Three polysaccharide fractions, NSP-1, NSP-2, and NSP-3, were isolated from *Nitraria sibirica*. Among them, NSP-3 exhibited the strongest antiproliferative effect against MCF-7 breast cancer cells with an IC_50_ value of 50.89 ± 2.75 μg/mL in a dose-dependent manner. Both NSP-1 and NSP-3 suppressed the proliferation of B-16 melanoma cells. All three fractions inhibited the growth of CT-26 colorectal adenocarcinoma cells, whereas BGC-823 gastric cancer cells displayed obvious inhibitory responses only to NSP-2 [15]. Experimental evidence indicates that *Nitraria tangutorum* polysaccharide NTWP-Ap exerts antitumor effects in a Lewis lung carcinoma (LLC) subcutaneous xenograft mouse model. Continuous intragastric administration at doses of 50 mg/kg and 100 mg/kg for 14 d retarded tumour growth. The tumour weight was significantly reduced in the 100 mg/kg group compared with the control group (*p* < 0.05), and tumour-tissue CD31 expression decreased in a dose-dependent fashion (*p* < 0.01). Both dosages markedly elevated the proportion of M1-type tumour-associated macrophages (TAMs) and lowered the percentage of M2-type TAMs (*p* < 0.01). Moreover, the proportion of CD8^+^ T cells increased while regulatory T-cell (Treg) numbers declined, contributing to the remodelling of the tumour-immune microenvironment. *In vitro* assays revealed that NTWP-Ap at a 100–400 μg/mL range promoted the polarization of RAW264.7 macrophages toward the M1 phenotype. Conditioned medium obtained from RAW264.7 cells treated with 200 μg/mL and 400 μg/mL NTWP-Ap significantly triggered LLC cell apoptosis (*p* < 0.01), which is presumed to be mediated via the TLR4/MAPK/NF-κB signalling pathway [18]. Collectively, *Nitraria* polysaccharides exert multi-targeted antitumor effects. They can directly repress proliferation of certain tumour cells and remodel the tumour microenvironment by promoting M1-type TAM polarization, enhancing CD8^+^ T-cell-mediated antitumour immunity, reducing Treg proportions and inhibiting tumour angiogenesis (Figure 5).

### 4.4. Hypoglycaemic Activity

**Figure 6:**
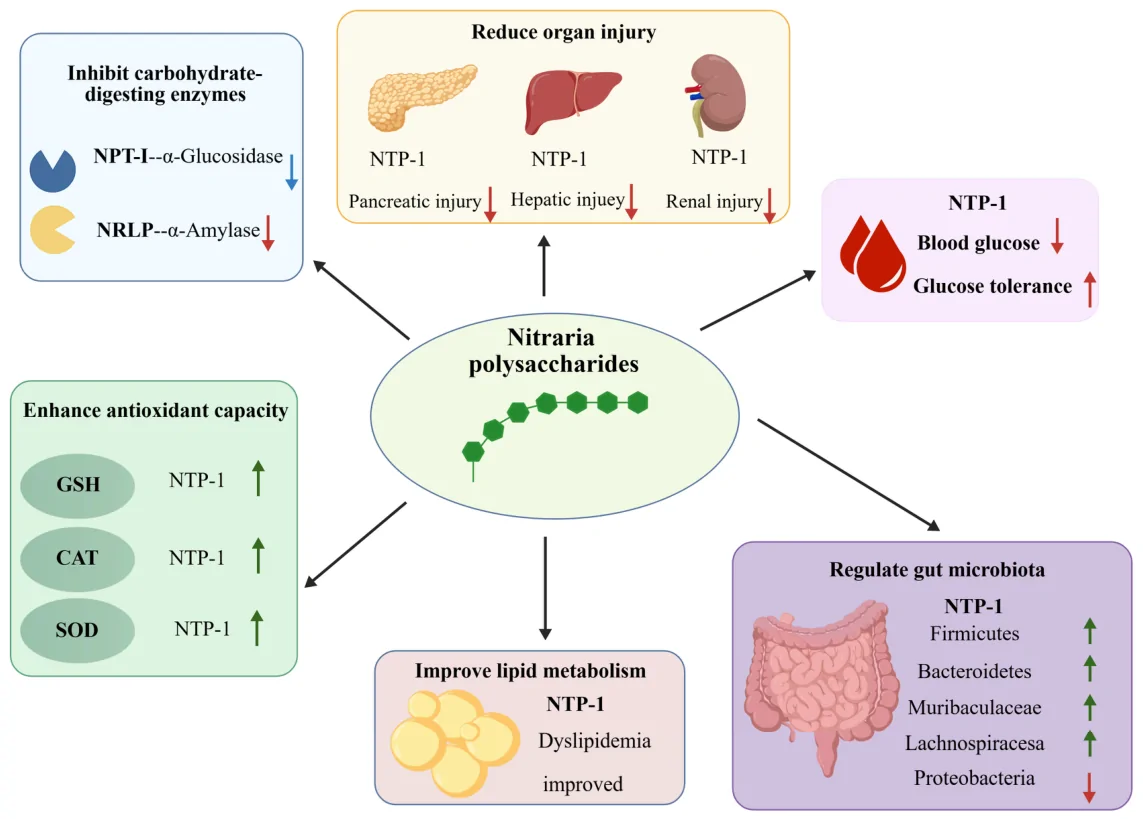
The potential hypoglycaemic activity of *Nitraria* L. polysaccharides. Upward arrows (↑) indicate an increase or upregulation, downward arrows (↓) indicate a decrease or downregulation, and connecting arrows indicate the proposed relationships or effects.

Hypoglycaemic bioactivities are manifested in lowering elevated blood-glucose levels, ameliorating glucose tolerance and disorders of glucose and lipid metabolism, as well as alleviating diabetes-related metabolic injuries via protecting islet function, boosting antioxidant capacity and modulating gut microbiota. A low-molecular-weight polysaccharide, NTP-I (molecular weight 9932 Da), was isolated from *Nitraria tangutorum*, which exhibited an α-glucosidase inhibitory rate of 84.3 ± 3.4%. In streptozotocin (STZ)-induced diabetic mice, 200 mg/kg was identified as the optimal intervention dosage. Compared with the model group, fasting blood glucose was reduced by 51.6%, glucose tolerance was improved by 38.2%, and the area under the glucose-tolerance curve decreased by 47.9%. This polysaccharide also ameliorated dyslipidaemia; increased the level of reduced glutathione (GSH), a major intracellular non-enzymatic antioxidant; and enhanced the activities of antioxidant enzymes including catalase (CAT) and superoxide dismutase (SOD). It also mitigated pathological injuries in the pancreas, liver, and kidney. Furthermore, it increased the relative abundance of Firmicutes, Bacteroidetes, Muribaculaceae, and Lachnospiraceae, while decreasing the relative abundance of Proteobacteria [21]. These alterations in gut microbial composition occurred together with reductions in fasting blood glucose and improvements in glucose tolerance and lipid metabolism, suggesting that modulation of the gut microbiota may contribute to the metabolic effects of NTP-I. However, the available evidence does not establish a direct causal relationship between individual bacterial taxa and the hypoglycaemic effect. Water-soluble leaf polysaccharide NRLP extracted from *Nitraria retusa* exerted dose-dependent inhibitory effects against α-amylase with an IC_50_ of 4.55 mg/mL, whereas the positive-control acarbose yielded an IC_50_ of 1.04 mg/mL. This result suggests that NRLP possesses an *in vitro* digestive-enzyme-inhibitory capacity and potential hypoglycaemic properties [24]. Studies at the whole-fruit level demonstrated that dosages of 1.8 g/kg and 3.6 g/kg could decrease blood-glucose levels in alloxan-induced diabetic mice, improve glucose tolerance in alloxan-induced diabetic rats, and counteract hyperglycaemia triggered by adrenaline and glucose, without altering blood-glucose concentrations in normal mice. The maximum intragastric dosage in acute-toxicity tests reached 53.5 g/kg [38]. *Nitraria* and its polysaccharides display favourable hypoglycaemic performance. Particularly, low-molecular-weight NTP-I can ameliorate diabetes-associated metabolic abnormalities through multiple pathways: lowering blood glucose, restoring glucose tolerance, regulating glucose–lipid metabolism, enhancing antioxidant defence, alleviating organ damage, and reshaping gut microbiota. Hence, it holds promising prospects for development as natural hypoglycaemic ingredients and functional-food raw materials (Figure 6).

### 4.5. Anti-Fatigue Activity

Anti-fatigue activities are reflected in enhancing exercise endurance, delaying fatigue onset, and accelerating post-exercise recovery. Such bioactivities are generally associated with sustaining energy reserves, reducing accumulation of fatigue-related metabolites such as lactic acid and blood urea nitrogen (BUN), and alleviating exercise-provoked oxidative damage [39]. At 50 mg/kg, water-soluble *Nitraria tangutorum* polysaccharide NTWP significantly shortened the immobility duration in the forced-swim test; more pronounced effects were observed at 100 mg/kg and 200 mg/kg (*p* < 0.01). At 200 mg/kg, NTWP decreased serum LDH from 847 ± 16.2 U/L to 599 ± 22.1 U/L and MDA from 22.5 ± 1.7 nmol/mL to 16.0 ± 1.1 nmol/mL. Meanwhile, it raised SOD activity from 96.8 ± 33.9 U/mL to 142.1 ± 18.2 U/mL and GPx activity from 495 ± 33 U/L to 647 ± 37 U/L, indicating that its anti-fatigue function is partly attributed to the mitigation of exercise-related oxidative injury [19]. Research shows that after treatment with high-dose (3 g/kg) and low-dose (1.5 g/kg) *Nitraria tangutorum* fruit polysaccharides, the weight-loaded exhaustive swimming time of mice was prolonged from 5.59 ± 0.76 min (control) to 7.82 ± 0.83 min and 6.42 ± 0.72 min, respectively. Serum lactic acid dropped from 6.59 ± 0.76 mmol/L to 3.82 ± 0.83 mmol/L and 4.42 ± 0.72 mmol/L; BUN decreased from 11.52 ± 1.64 mmol/L to 8.19 ± 1.57 mmol/L and 7.25 ± 1.94 mmol/L. Hepatic glycogen content increased from 12.96 ± 0.88 mg/g to 16.43 ± 0.97 mg/g and 14.61 ± 0.81 mg/g, demonstrating improved exercise endurance and reduced accumulation of fatigue metabolites [34]. In summary, the reported anti-fatigue effects of *Nitraria* polysaccharides were associated with prolonged exercise tolerance, reduced levels of fatigue-related metabolites such as lactic acid and blood urea nitrogen (BUN), increased glycogen storage, enhanced antioxidant-enzyme activities, and reduced markers of exercise-induced oxidative injury.

### 4.6. Anti-Stress Activity

Anti-stress activity refers to the capacity to maintain physiological homeostasis and improve organismal tolerance under adverse environmental stimuli such as high temperature, low temperature, and hypoxia. External stress disrupts body homeostasis, triggering metabolic disturbance and physiological dysfunction. Accordingly, improving adaptability to extreme-environment and hypoxic conditions bears great practical significance [5,32]. Studies have evaluated the anti-stress effects of *Nitraria tangutorum* fruit polysaccharides using high-temperature (45 °C), low-temperature (−5 °C), and normobaric-hypoxia mouse models [34]. After administration of high-dose (3 g/kg) and low-dose (1.5 g/kg) polysaccharides, mouse survival time under 45 °C heat stress reached 144.1 ± 29.39 min and 139.4 ± 5.98 min, longer than the 113.3 ± 5.37 min observed in the control group. Under −5 °C cold exposure, survival time was extended from 105.3 ± 8.45 min (control) to 120.0 ± 21.32 min and 118.4 ± 18.54 min (*p* < 0.01). Under normobaric hypoxia, survival times for high-dose and low-dose groups were 65.7 ± 11.3 min and 62.5 ± 7.9 min, markedly higher than the control value of 55.6 ± 8.4 min (*p* < 0.05 and *p* < 0.01, respectively). These results demonstrate that *Nitraria tangutorum* polysaccharides enhance mouse tolerance to diverse environmental stresses, particularly prolonging survival under high-temperature, low-temperature, and hypoxic challenges. Nevertheless, current anti-stress investigations on *Nitraria* polysaccharides are mainly confined to phenotypic indices such as animal survival time; their precise mechanisms and structure–activity relationships require further exploration. These findings provide phenotypic evidence of enhanced stress tolerance, but survival time alone does not reveal the underlying mechanism. Potential mechanisms may involve maintenance of energy homeostasis, attenuation of stress-induced oxidative damage, and regulation of cellular responses to hypoxic and thermal stress. However, these pathways have not been directly demonstrated for the *Nitraria* tangutorum polysaccharide preparation used in the reported study. Future investigations should therefore combine survival endpoints with measurements of energy-metabolism indices, oxidative-stress biomarkers, antioxidant-enzyme activities, and molecular markers associated with hypoxic and temperature-stress responses.

### 4.7. Hepatoprotective Activity

Hepatoprotective activity is characterised by alleviating hepatocyte injury, decreasing serum alanine aminotransferase (ALT) and aspartate aminotransferase (AST) levels, and restoring hepatic tissue function. In a CCl_4_-induced acute chemical liver-injury mouse model, after 15 consecutive days of drug administration, serum ALT levels in high- and low-dose groups of *Nitraria tangutorum* fruit polysaccharides were 71.4 ± 17.8 U/mL and 82.9 ± 14.6 U/mL, obviously lower than 108.5 ± 13.6 U/mL in the model group. Corresponding AST values were 55.4 ± 17.8 U/mL and 68.9 ± 14.6 U/mL, also lower than the model-group value of 78.5 ± 13.6 U/mL, which confirms that the polysaccharides can relieve CCl_4_-provoked hepatocyte damage [34]. At present, research concerning the hepatoprotective properties of *Nitraria* polysaccharides remains largely based on animal experiments. Their exact molecular targets and structure–activity relationships still need further clarification. Existing findings provide experimental evidence for their application in liver-injury protection and the development of related functional products.

**Table 4:** Bioactivity of *Nitraria* L. polysaccharides.

Number	Designation of Polysaccharides	Experimental System/Model	Dose/Concentration (Route)	Bioactivity	References
1	NTWP-II	*In vitro*; DPPH, hydroxyl-radical and superoxide-anion-radical scavenging assays; ferrous-ion chelation assay	0.02–5.0 mg/mL (N/A)	Antioxidant activity; active groups in the molecular structure donate electrons or hydrogen atoms to terminate free-radical chain reactions, while moderate molecular weight and high hydrogen-donating capacity enhance antioxidant activity	[27]
2	NTP-I	*In vitro*; DPPH-radical scavenging and α-glucosidase-inhibition assays	NR (N/A)	Antioxidant activity; the DPPH-radical scavenging rate was 71.72 ± 2.1%, and the α-glucosidase inhibition rate was 84.3 ± 3.4%; antioxidant effects were exerted through active functional groups	[21]
3	NSP-C	*In vitro*; DPPH and hydroxyl-radical scavenging assays	Multiple concentrations; 2.0 mg/mL explicitly reported (N/A)	Antioxidant activity; the IC_50_ values for DPPH and hydroxyl-radical scavenging were 0.209 and 0.149 mg/mL, respectively, showing dose-dependent activity	[14]
4	NRFP-1, NRFP-2, NRFP-3	*In vitro*; DPPH, ABTS•+ radical-cation, and hydroxyl-radical scavenging assays; Fe^3+^-reducing-power assay	0.2, 0.4, 0.6, 0.8, and 1.0 mg/mL (N/A)	Antioxidant activity; related to arabinose and mannose contents and molecular weight; free radicals are scavenged through hydrogen or electron donation, lipid peroxidation is inhibited, and the activity is concentration dependent	[22]
5	Crude NSP	*In vitro*; DPPH, ABTS, and hydroxyl-radical scavenging assays	Up to 1.0 mg/mL (N/A)	Antioxidant activity; radicals were scavenged in a concentration-dependent manner, with IC_50_ values of 0.183 mg/mL for ABTS and 0.604 mg/mL for hydroxyl radicals.	[15]
6	CNRFP	*In vitro*; DPPH, ABTS, and hydroxyl-radical scavenging assays; reducing-power assay	NR (N/A)	Antioxidant activity; the IC_50_ values for DPPH, ABTS, and hydroxyl radicals were 1.14, 0.54, and 1.11 mg/mL, respectively; reducing power increased with concentration, and hydroxyl groups and uronic-acid carboxyl groups contributed to radical quenching and metal-ion chelation	[32]
7	NRFP	*In vitro*; total antioxidant-capacity, DPPH, H_2_O_2_-scavenging, and H_2_O_2_-induced erythrocyte-hemolysis assays	2–12 mg/mL (total antioxidant capacity); 0.25–4 mg/mL (DPPH); 0.1–5 mg/mL (H_2_O_2_); 10–250 μg/mL (hemolysis) (N/A)	Antioxidant activity; hydroxyl and other functional groups scavenge free radicals, inhibit lipid peroxidation, protect erythrocyte membrane integrity, and exhibit concentration-dependent activity	[23]
8	NRLP	*In vitro*; total antioxidant-capacity, DPPH, and hydroxyl-radical scavenging assays	0.5, 1.0, 1.5, 2.0, 2.5 and 3.0 mg/mL (N/A)	Antioxidant activity; the IC_50_ values were 2.03 mg/mL for total antioxidant capacity, 2.7 mg/mL for DPPH scavenging, and 246.85 μg/mL for hydroxyl-radical scavenging	[24]
9	NTP, CMNTP	*In vitro*; hydroxyl-radical, superoxide-anion-radical, and DPPH scavenging assays	Hydroxyl radical: multiple concentrations, with a 0.05–0.40 mg/mL range explicitly discussed; superoxide anion: 0.2–1.0 mg/mL; DPPH: 0.05–1.0 mg/mL (N/A)	Antioxidant activity; both polysaccharides scavenged all three types of radicals, while carboxymethylation enhanced hydroxyl-radical and superoxide-anion scavenging; antioxidant effects were achieved through radical capture by active molecular groups	[17]
10	*Nitraria tangutorum* polysaccharides	*In vitro*; DPPH and hydroxyl-radical scavenging assays; reducing-power assay	NR (N/A)	Antioxidant activity; scavenged DPPH and hydroxyl radicals and exhibited reducing capacity; active hydrogen atoms terminated free-radical chain reactions	[28]
11	*Nitraria tangutorum* polysaccharides	*In vivo*; mouse free-swimming oxidative-stress model; serum SOD and MDA determination	1.5 and 3 g/kg (route NR)	Antioxidant activity; significantly increased SOD activity and decreased MDA content by enhancing endogenous antioxidant-enzyme activity and inhibiting lipid peroxidation	[34]
12	Crude *Nitraria* polysaccharides	*In vivo*; progressive-load aerobic-exercise model in aged rats; CAT, GSH-Px, SOD, and MDA determination	100, 200 and 300 mg/(kg·d) (oral)	Antioxidant activity; increased myocardial CAT, GSH-Px, and SOD activities; decreased MDA content; and reduced skeletal-muscle lipofuscin accumulation and cell apoptosis	[35]
13	BCH	*In vitro*; superoxide-anion, DPPH, and hydroxyl-radical scavenging assays; reducing-power assay	NR (N/A)	Antioxidant activity; scavenged free radicals, with EC_50_ values below 0.1 mg/mL for superoxide anion and DPPH radicals	[36]
14	NTP, CMNTP	*In vivo*; cyclophosphamide-induced immunosuppressed mouse model	100 and 200 mg/kg (oral gavage)	Immunomodulatory activity; increased thymus and spleen indices, elevated serum IFN-γ and IL-4 levels, improved Th1/Th2 immune imbalance, increased intestinal villus length, attenuated the reduction in the V/C ratio, and promoted intestinal SIgA secretion; carboxymethylation further enhanced the activity	[17]
15	NTB	*In vivo*; cyclophosphamide-induced immunosuppressed mouse model	3.6 g/kg (route NR)	Immunomodulatory activity; increased thymus and spleen indices, improved splenic histological structure, elevated serum IFN-γ levels and splenic IFN-γ mRNA expression, and increased CD3^+^, CD4^+^, CD8^+^ T cells, and NK cells	[37]
16	NSP-1, NSP-2, NSP-3	*In vitro*; COX-2 enzyme-inhibition assay	100 μg/mL (N/A)	Anti-inflammatory activity; at 100 μg/mL, the COX-2 inhibition rate of NSP-3 reached 85.6%, higher than that of NSP-1 (79.6%) and NSP-2 (54.7%)	[15]
17	NRLP	*In vivo*; carrageenan-induced paw-oedema model	100 and 400 mg/kg bw (oral)	Anti-inflammatory activity; inhibited the release of inflammatory mediators such as histamine and prostaglandins, decreased MDA content, and alleviated lipid-peroxidation injury	[24]
18	NSP-1, NSP-2, NSP-3	*In vitro*; proliferation assays using MCF-7, B-16, CT-26, and BGC-823 tumour cells	100 and 1000 μg/mL for B-16, CT-26 and BGC-823 assays; MCF-7 concentration series NR (N/A)	Antitumor activity; inhibited MCF-7 cell proliferation with an IC_50_ of 50.89 μg/mL; the high uronic-acid content was associated with enhanced activity	[15]
19	NTWP-Ap	*In vivo/in vitro*; LLC subcutaneous tumour-bearing mouse model; *in vitro* RAW264.7 macrophage-polarization assay	*In vivo*: 50 and 100 mg/kg (oral gavage); *in vitro*: 100, 200, and 400 μg/mL (N/A)	Antitumor activity; induced macrophage polarization toward the M1 phenotype through the TLR4/MAPK/NF-κB signalling pathway and promoted TNF-α, IL-6, and IL-1β secretion; *in vivo*, it increased M1-type TAMs, decreased M2-type TAMs, enhanced CD8^+^ T-cell infiltration, reduced Treg cells, and downregulated CD31 expression to inhibit intratumoral angiogenesis	[18]
20	NTP-I	*In vivo*; STZ-induced diabetic mouse model	100, 200, and 300 mg/kg bw (oral gavage)	Hypoglycaemic activity; 200 mg/kg·bw was the optimal dose; reducing fasting blood glucose by 51.6%; improving glucose and lipid metabolism; repairing pancreatic, hepatic, and renal injury; enhancing antioxidant-enzyme activity; modulating gut microbiota; and increasing the abundance of beneficial bacteria	[21]
21	Lyophilized *Nitraria tangutorum* fruit powder	*In vivo*; alloxan-induced diabetic mouse/rat models and adrenaline-/glucose-induced hyperglycaemic mouse models	1.8 and 3.6 g/kg (route NR)	Hypoglycaemic activity; exerted protective and restorative effects on pancreatic β-cell injury and improved glucose tolerance	[38]
22	NRLP	*In vitro*; α-amylase-inhibition assay	0.5, 1, 3, 6, 9, and 12 mg/mL (N/A)	α-Amylase inhibitory activity; inhibited α-amylase in a concentration-dependent manner with an IC_50_ of 4.55 mg/mL, thereby potentially delaying carbohydrate digestion	[24]
23	NTWP	*In vivo*; mouse forced-swimming test	50, 100, and 200 mg/kg (oral)	Anti-fatigue activity; exerted anti-fatigue effects through regulation of energy metabolism and protection against oxidative injury, reduced fatigue-related biomarkers, and improved exercise endurance	[19]
24	*Nitraria tangutorum* polysaccharides	*In vivo*; weight-loaded swimming and free-swimming tests; serum lactic acid, BUN, and hepatic-glycogen determination	1.5 and 3.0 g/kg (route NR)	Anti-fatigue activity; prolonged exhaustive swimming time (*p* < 0.01), decreased serum lactic acid (*p* < 0.01) and BUN levels, and increased hepatic glycogen content (*p* < 0.01)	[34]
25	*Nitraria tangutorum* polysaccharides	*In vivo*; high-temperature (45 °C), low-temperature (−5 °C), and normobaric-hypoxia mouse models	1.5 and 3 g/kg (route NR)	Anti-stress activity; prolonged survival under extreme environmental conditions and enhanced organismal stress tolerance	[34]
26	*Nitraria tangutorum* polysaccharides	*In vivo*; CCl_4_-induced acute liver-injury mouse model; serum ALT and AST determination	NR (route NR)	Hepatoprotective activity; attenuated CCl_4_-induced hepatocyte injury, decreased serum ALT and AST activities, maintained hepatocyte membrane integrity, and alleviated chemical liver injury	[34]
27	NRFP	*In vivo*; Triton X-100-induced hyperlipidaemic mouse model	250 and 500 mg/kg (p.o.)	Hypolipidemic activity; decreased serum TC, TG, and LDL-C levels; increased HDL-C levels; reduced the atherosclerosis index, cardiac index, and coronary artery index; and alleviated lipid-metabolism disorders	[23]
28	NRLP	*In vivo*; hot-plate, acetic-acid-induced writhing, and formalin-induced pain models	100 and 400 mg/kg (p.o.)	Analgesic activity; at 400 mg/kg, inhibited formalin-induced pain with an inhibition rate of 76.4% during the inflammatory phase and exhibited both central and peripheral analgesic effects	[24]
29	NTP	*In vivo*; LPS-induced acute lung-injury mouse model	200 and 400 mg/kg/day (intragastric)	Protective activity against acute lung injury; at 200–400 mg/kg, NTP reduced pulmonary oedema and inflammatory-cell infiltration and decreased TNF-α, IL-1β, and IL-6 levels; its protective effect was proposed to be associated with suppression of the TLR4/IKK/NF-κB signalling pathway.	[20]
30	NTP-L	*In vivo*; healthy C57BL/6J mice; intestinal morphology, gut-microbiota, and faecal-SCFA analyses	200 mg/kg (route NR)	Gut-microbiota-modulating activity; increased ileal villus length and improved intestinal mucosal structure; increased faecal SCFA contents, including acetic acid, butyric acid, valeric acid, isobutyric acid, and isocaproic acid; enriched potentially beneficial taxa such as Rikenellaceae_RC9_gut_group and Lachnospiraceae_NK4A136_group	[40]

Note: NR, not reported; N/A, not applicable; p.o., oral administration. Doses and concentrations are reported as provided in the original studies. Owing to differences in experimental systems, *in vitro* concentrations and *in vivo* doses are not directly comparable.

### 4.8. Hypolipidaemic Activity

Lipid-modulating effects are reflected in changes in serum total cholesterol (TC), triglycerides (TGs), low-density lipoprotein cholesterol (LDL-C), and high-density lipoprotein cholesterol (HDL-C), together with reductions in lipid deposition and oxidative damage under hyperlipidaemic conditions. In a Triton X-100-induced hyperlipidaemic mouse model, mice were treated with NRFP at 250 mg/kg and 500 mg/kg for seven consecutive days [23]. In the model group, compared with normal controls, TC, TG, and LDL-C rose by 68.18%, 72.47%, and 41.48%, while HDL-C decreased by 36.48%. After NRFP intervention, TC, TG, and LDL-C were significantly reduced and HDL-C was markedly elevated (*p* < 0.01). At 500 mg/kg, NRFP reduced coronary-artery index (CAI), cardiac index (CI), and atherosclerosis index (AI) by 39.51%, 41.74%, and 59.63%, respectively. Furthermore, 250 mg/kg and 500 mg/kg NRFP decreased hepatic MDA content by 36.34% and 55.25%, and cardiac MDA by 17.43% and 45.10%, suggesting that NRFP ameliorates dyslipidaemia and mitigates hyperlipidaemia-associated lipid-peroxidation injury. To date, studies on the lipid-regulating capacity of *Nitraria* polysaccharides are still limited. Their specific regulatory targets as well as structure–activity relationships merit further research.

### 4.9. Other Biological Activities

Apart from the above-mentioned bioactivities, *Nitraria* polysaccharides also exert analgesic, organ-protective, and gut-microbiota-modulating functions. Studies have reported that water-soluble leaf polysaccharide NRLP from *Nitraria retusa* produced dose-dependent α-amylase-inhibitory activity with an IC_50_ of 4.55 mg/mL [24]. In the acetic-acid writhing test, pain-inhibition rates reached 28.6% and 50.6% at dosages of 100 mg/kg and 400 mg/kg, respectively. At 400 mg/kg, the inhibitory rates against formalin-evoked neurogenic and inflammatory pain attained 60.0% and 76.4%, demonstrating favourable analgesic potential. In an LPS-induced acute lung-injury mouse model, 21-day oral administration of *Nitraria tangutorum* polysaccharide NTP (200 mg/kg and 400 mg/kg) alleviated pulmonary oedema, inflammatory-cell infiltration, and thickening of alveolar septa. It also lowered TNF-α, IL-1β, and IL-6 levels; increased SOD activity; and reduced MDA accumulation. Its lung-protective effect is presumed to be linked to the suppression of the TLR4/IKK/NF-κB signalling pathway [20].

Moreover, investigations indicate that 14-day intervention with low-molecular-weight *Nitraria tangutorum* polysaccharide NTP-L (200 mg/kg) increased mouse ileal villus height from 288.41 ± 18.57 μm to 315.13 ± 15.27 μm (*p* < 0.05). It improved gut-microbiota richness and diversity and enriched potential beneficial taxa such as Rikenellaceae_RC9_gut_group and Lachnospiraceae_NK4A136_group. Faecal acetic-acid and butyric-acid contents rose from 1.94 ± 0.38 μg/mg to 2.84 ± 0.25 μg/mg and from 0.221 ± 0.159 μg/mg to 0.680 ± 0.168 μg/mg, respectively (*p* < 0.05). These observations reveal its capacity to ameliorate intestinal–mucosal architecture and modulate gut microecology [40]. Polysaccharides can be fermented by intestinal microbes to reshape microbiota composition and metabolic profiles, implying that gut microbiota may serve as an important mediator for the physiological regulatory functions of *Nitraria* polysaccharides [41].

Comprehensive analysis of domestic and international research reports concerning the biological activities of *Nitraria* polysaccharides reveals that NMR-based structural characterization has been performed on two polysaccharides from *Nitraria tangutorum*, with their antioxidant and hypoglycaemic activities well-documented. Four publications have investigated the glycosidic-linkage patterns of *Nitraria* polysaccharides covering *Nitraria tangutorum*, *Nitraria sibirica*, and *Nitraria retusa*, alongside evaluations of their anti-fatigue, antioxidant, and lipid-regulating activities, respectively. Although existing studies have verified multiple bioactivities including anti-fatigue, anti-stress, hepatoprotective, immunomodulatory, and hypoglycaemic properties, several limitations remain. Most bioactivity investigations merely validate the overall biological effects of crude or purified polysaccharide fractions. Research regarding the in vivo digestion and fermentation behaviours of *Nitraria* polysaccharides is generally scarce. Moreover, mechanistic explorations for various bioactivities remain superficial. In-depth dissection of regulatory signalling pathways and molecular targets in combination with gut microbiota and other *in vivo* microecosystems is largely absent. Certain activity-related studies also lack correlative analyses linking bioactivity to *in vivo* metabolism and tissue-organ responses, resulting in a relatively simplistic research framework.

## 5. Conclusions and Future Perspectives

As the core bioactive constituents derived from *Nitraria*, a medicinal-and-edible plant indigenous to the desert regions of Northwest China, *Nitraria* polysaccharides have been verified to exert multiple pharmacological properties including antioxidant, immunomodulatory, anti-fatigue, hypoglycemic, and antitumor activities, which endow them promising development prospects in the fields of functional foods and natural medicines. This review systematically summarizes the research progress concerning the extraction-preparation, structural characterization, and biological activities of *Nitraria* polysaccharides. The effects of diverse extraction protocols on polysaccharide yield are clarified. The structural diversity of these heteropolysaccharides is concluded in terms of molecular weight, monosaccharide composition, and glycosidic-bond types. Furthermore, the multi-target pharmacological mechanisms are generalized, including radical scavenging, activation of immune signalling pathways, and gut-microbiota modulation. Nevertheless, several bottlenecks still restrict current investigations. Structural characterizations are mostly limited to primary structures, whereas higher-order conformations (e.g., helical and chain conformations) are rarely explored. Most pharmacological experiments adopt crude polysaccharide mixtures; the *in vivo* metabolic behaviours and molecular targets of homogeneous polysaccharides remain to be systematically elucidated. The laboratory-scale purification strategy based on DEAE ion-exchange chromatography suffers from high cost and limited loading capacity, and industrial-scale production technologies are still absent. Additionally, whether the native saline–alkali habitat of *Nitraria* alters polysaccharide biosynthesis and bioactivities via alkali-stress responses has drawn little research attention. In future studies, multi-dimensional chromatography–mass spectrometry hyphenated techniques, NMR, atomic force microscopy (AFM), and small-angle X-ray scattering (SAXS) should be utilized to deeply decipher the higher-order conformations of *Nitraria* polysaccharides. Multi-omics approaches including transcriptomics, proteomics, and metabolomics need to be integrated to systematically clarify their multi-target pharmacological mechanisms. Techniques such as structural modification and nano-delivery systems can be adopted to improve their bioavailability. Moreover, safety evaluation and standardization research should be further perfected, so as to promote the in-depth exploitation and commercial application of *Nitraria* polysaccharides as raw materials for functional foods or natural medicines.

## Figures and Tables

**Table 1:** Raw materials investigated in studies of *Nitraria* polysaccharides.

Species	Geographic Origin	Plant Organ	Polysaccharide Studied	Reference
*Nitraria sibirica* Pall.	Hetian, Xinjiang, China	Fruit	NSP-C; crude NSP and NSP-1, NSP-2, and NSP-3	[14,15]
*Nitraria sibirica* Pall.	Mongolian Gobi Desert, Mongolia	Green leaves	Pectic polysaccharide NS	[16]
*Nitraria tangutorum* Bobr.	Qinghai, China, mainly the Qaidam Basin/Haixi region	Fruit	NTP/CMNTP; NTWP and its fractions; NTP	[17,18,19,20]
*Nitraria tangutorum* Bobr.	Wuwei, Gansu, China	Fruit	Crude NTP; NTP-I and NTP-II	[21]
*Nitraria retusa*	Baicheng County, Xinjiang, China	Fruit	Crude NRFP; NRFP-1, NRFP-2, and NRFP-3	[22]
*Nitraria retusa*	Tabeddit, Gafsa, Tunisia	Fruit/leaves	NRFP (fruit); NRLP (leaves)	[23,24]
*Nitraria* species	Coastal area of Cangzhou, Hebei, China	Fruit	Water-soluble fruit polysaccharide	[25]

**Table 2:** Study of the extraction of *Nitraria* L. polysaccharides.

Extraction Methods	Designation of Polysaccharides	Theory	Key Parameters	Yield (%)	Benefits and Limitations	References
Hot-Water Extraction	NTP, NTWP-II, NTWP-Ap, NTWP, NRFP, NRLP	Taking advantage of the water solubility of polysaccharides, high temperatures are used to promote their leaching from plant cells	Extraction temperatures (60–100 °C; 79 °C, 94 °C, 60 °C, 90 °C, 80 °C, 100 °C), solid-to-liquid ratios (1:15, 1:5, 15:1 mL/g, 1:20, 1:10), extraction times (1.5 h, 2 h, 7 h, 5 h, 6 h), and number of extraction cycles (two, three, or as otherwise reported in the individual studies)	NTP: 39.67 ± 0.02; NTWP-II: 14.01 ± 0.11; NTWP-Ap: 4.68; NTWP: 4.80; water-soluble polysaccharide: ≈1.0; NRFP: 8.65; NRLP: ≈4.00; NTP: ≈3.59; NR	Simple operation, low cost, and no reagent residues; however, the process involves long extraction times and high energy consumption, and some polysaccharides may degrade due to high temperatures	[17,18,19,20,23,24,25,26,27]
Enzyme-Assisted Extraction	NSP-C, NTP, CMNTP	Use cellulases, pectinases, and other enzymes to break down plant cell-wall structures and promote the release of polysaccharides	Enzyme dosages (0.5% cellulase; 2.5% total pectinase/cellulase), pH (5.5; 3.0), extraction temperatures (50 °C; 40 °C), extraction times (2 h; 58 min)	NSP-C: 13.82; NTP: 39.57 ± 0.11	Low extraction temperature, short extraction time, and low energy consumption; increased costs, and the need to control pH and temperature	[14,17]
Ultrasound-Assisted Extraction	NTP-I, NRFP, NRFP-1, NRFP-2, NRFP-3	The ultrasonic cavitation effect disrupts cell structures, accelerating the dissolution of polysaccharides	Ultrasonic power (200 W; NR), extraction temperatures (70 °C; 59.5 °C), solid-to-liquid ratio (1:15; 19.5 mL/g), and times (20 min ultrasonication followed by 1.5 h water-bath extraction; 30.5 min)	NTP: 5.60; NRFP: 3.35 ± 0.12	High extraction efficiency, short processing time, and relatively low energy consumption; high equipment costs	[21,22]
Accelerated Solvent Extraction	Crude *Nitraria tangutorum* polysaccharide	Enhances solvent penetration under high temperature and pressure for rapid and efficient polysaccharide extraction	Temperature (102 °C), pressure (10 MPa), time (10 min per cycle), and number of extraction cycles (two)	NTP: 78.15	High extraction yield, fast speed, and low solvent consumption; high equipment costs; high temperature and pressure may damage the activity of polysaccharides	[15]
Ultrasound-Enzyme-Assisted	NSP-1, NSP-2, NSP-3	Utilizes the ultrasonic cavitation effect and enzymes such as cellulase and pectinase to break down plant cell structures and promote the release of polysaccharides	Enzyme type (cellulase), enzyme concentration (0.5%), feed-to-liquid ratio (1:20), pH (5.5), temperature (50 °C), time (50 min), and ultrasonic power (500 W)	NSP: 12.55 ± 1.12	High extraction efficiency; high cost, strict control of process parameters, and challenges in large-scale production	[28]
Sequential Water/Chelating Extraction	NS pectic polysaccharide	Weakly acidic ammonium oxalate disrupts protopectin–cell-wall interactions and promotes the release of pectic polysaccharides	Temperatures (70 °C for water extraction, 50 °C for acid pretreatment, and 70 °C for ammonium oxalate extraction) and ammonium oxalate concentration (0.7%, *w*/*v*; acid pretreatment at pH 4.0)	NS: 2.0	Ammonium oxalate extraction can elute bound pectin and achieves a higher extraction yield, but it tends to co-extract protein impurities and may slightly alter the pectin structure, making the process more complex	[16]

**Table 3:** Summary of structural features of *Nitraria* L. polysaccharides.

Number	Source (Species, Origin, Organ)	Designation of Polysaccharides	Separation and Purification Methods	Molecular Weight (Da)	Monosaccharide Composition	Glycosidic-Linkage Pattern	References
1	*Nitraria tangutorum*; Gansu Province; Fruit	NTWP-II	DEAE-52 G-200	229,000	Rha:Ara:Man:Glc:Gal = 1.14:2.5:3.00:2.69:5.28	→4)-α-L-Araf-(1→ →3)-β-D-Galp-(1→ →6)-α-L-Rhap-(1→ α-D-Glcp-(1→ α-D-Manp-(1→	[27]
2	*Nitraria tangutorum*; Gansu Province; Fruit	NTP-I	DEAE-52	9932	Ara:Gal:Man:Glc:Xyl:Fru = 10.78:4.76:20.86:20.36:2.56:13.4	→2)-α-D-Manp-(1→ →4)-β-D-Xylp-(1→ →4,6)-β-D-Glcp-(1→ α-D-Galp-(1→	[21]
3	*Nitraria sibirica*; Xinjiang Uygur Autonomous Region; Fruit	NSP-C	/	5070	Rha:Ara:Man:Glc:Gal = 1:1:0.73:2.04:3.75	-	[14]
4	*Nitraria retusa*; Xinjiang Uygur Autonomous Region; Fruit	NRFP-1	DEAE Sepharose Fast Flow	20,010	Man:Rib:Rha:GluA:GalA:Glc:Gal:Xyl:Ara:Fuc = 3.73:2.01:0.46:2.97:0.54:47.22:15.97:4.06:21.28:1.51	-	[22]
5	*Nitraria retusa*; Xinjiang Uygur Autonomous Region; Fruit	NRFP-2	DEAE Sepharose Fast Flow	28,960	Man:Rib:Rha:GalA:Glc:Gal:Xyl:Ara:Fuc = 3.93:0.30:1.24:3.03:2.95:8.05:9.26:6.10:43.07:0.30	-	[22]
6	*Nitraria retusa*; Xinjiang Uygur Autonomous Region; Fruit	NRFP-3	DEAE Sepharose Fast Flow	67,450	Man:Rib:Rha:GluA:GalA:Glc:Gal:Xyl:Ara:Fuc = 6.96:0.90:0.47:3.41:3.40:46.33:3.16:21.30:0.10	-	[22]
7	*Nitraria sibirica*; Xinjiang Uygur Autonomous Region; Fruit	NSP-1	DEAE-52	21,500	Man:Rha:Glc:Gal:Ara = 1.66:0.18:1.27:1.00:0.10	-	[15]
8	*Nitraria sibirica*; Xinjiang Uygur Autonomous Region; Fruit	NSP-2	DEAE-52	14,400	Man:Rha:Glc:Gal = 0.21:1.57:0.42:1.00	-	[15]
9	*Nitraria sibirica*; Xinjiang Uygur Autonomous Region; Fruit	NSP-3	DEAE-52	49,400	Rha:Glc:Gal = 3.21:0.64:1.00	-	[15]
10	*Nitraria tangutorum*; Qinghai Province; Fruit	NTP	-	74,359–438,665	Man:GlcUA:GalUA:Rha:Glc:Gal:Ara = 3.0:3.7:9.5:14.5:7.0:73.0:50.6	-	[17]
11	*Nitraria tangutorum*; Qinghai Province; Fruit	CMNTP	-	11,459–80,385	Man:GlcUA:GalUA:Rha:Glc:Gal:Ara = 7.3:4.5:13.7:15.1:2.6:52.5:37.1	-	[17]
12	*Nitraria tangutorum*; Qinghai Province; Fruit	NTWP-Ap	DEAE Sepharose Fast Flow	106,320	Ara:GalUA:Gal:Xyl = 58.92:30.19:7.49:3.40	-	[18]
13	*Nitraria tangutorum*; Qinghai Province; Fruit	NTWP	DEAE Cellulose	>1,000,000, 79,700, <10,000	Man:Rha:GlcUA:GalUA:Glc:Gal:Ara = 3.9:1.8:0.2:3.3:70.6:7.6:13.1	-	[19]
14	*Nitraria tangutorum*; Qinghai Province; Fruit	NTP	-	207,800, 40,800, 8490, 2730, 1490	Man:Rha:GalUA:Glc:Gal:Ara = 3.52:15.08:10.00:26.73:38.08:6.59	-	[20]
15	*Nitraria* species; Hebei Province; Fruit	*Nitraria* fruit water-soluble polysaccharides	-	-	Man:Rha:GalA:Glc:Gal:Ara = 9.2:3.3:1.1:1.0:1.9:2.3	-	[25]
16	*Nitraria retusa*; Gafsa; Fruit	NRFP	-	66,500	Glc:GalA:Gal:Ara:Rha = 41.4:30.5:12.6:11.8:3.70	→3)-β-D-Glcp-(1→ →3,6)-β-D-Glcp-(1→ →4)-β-D-GalpA-(1→ →2)-α-L-Rhap-(1→ →2,4)-α-L-Rhap-(1→ →4)-β-D-Galp-(1→ →5)-α-L-Araf-(1→ →2,5)-α-L-Araf-(1→ →3,5)-α-L-Araf-(1→	[23]
17	*Nitraria retusa*; Gafsa; Leaf	NRLP	-	23,060	Rha:Gal:GalA:Glc:Ara:Xyl:GlcA = 33.66:18.05:15.03:13.34:13.30:3.79:2.83	/	[24]

## Data Availability

The original contributions presented in this study are included in the article. Further inquiries can be directed to the corresponding author.
